# T144. THE ROLE OF TRANSIENT BETA OSCILLATIONS IN ABERRANT SELECTIVE ATTENTION TO SALIENT EVENTS IN SCHIZOPHRENIA

**DOI:** 10.1093/schbul/sby016.420

**Published:** 2018-04-01

**Authors:** Elizabeth Liddle, Jyothika Kumar, Siân Robson, Emma Hall, Lauren Gascoyne, Mohammad Katshu, Lena Palaniyappan, Peter Morris, Matthew Brookes, Peter Liddle

**Affiliations:** 1 University of Nottingham; 2 University of Western Ontario

## Abstract

**Background:**

Selective attention to situationally salient information is aberrant in schizophrenia. Following the presentation of behaviourally relevant stimuli, oscillatory power in the beta-band (13-30Hz) typically decreases (Event-Related Desynchronisation – ERD) then increases (Event-Related Synchronisation – ERS). The ERD-ERS pattern is a potential marker for the processing of behaviourally salient events. In a previous magnetoencephalography (MEG) study (Liddle et al Hum. Brain Mapp. 2016; 37:1361–74) we found that in people with schizophrenia, ERS was reduced. Recently, Jones (Curr. Opin. Neurobiol, 2016; 40: 72–80) proposed that the relatively continuous beta-synchronisation observed in trial-averaged data may reflect the probability distribution of transient beta events discernible in single trial data. She cited both animal and human data consistent with a neural model in which these beta bursts are generated by transient input to pyramidal neurons via distal dendrites concurrent with input to deeper layers presumed to be from thalamus. External stimuli are less likely to be perceived during the time period immediately following a transient beta event. The model is consistent with the hypothesis that transient beta bursts are an index of top-down modulation of the processing of perceptual information, and raises the possibility that aberrant control over this modulation might contribute to aberrant selective attention in schizophrenia. We hypothesized that in relevant trials, the beta-burst probability distribution would be skewed towards the latter part of the trial, reflecting a period of suppressed beta-burst probability, and thus of enhanced stimulus perception, followed by a period of increased burst probability, possibly reflecting sensory suppression following stimulus processing.

**Methods:**

We recorded MEG data in 23 patients with schizophrenia and 37 healthy controls during the performance of a relevance modulation task designed to assess neural effects of situational salience. Data were recorded using a 275-channel CTF system (Coquitlam, Canada). Visual stimuli that were either task-relevant or task-irrelevant were presented in alternating, predictable, order. Beamformed data time courses were computed for 8 previously defined brain networks. Time-frequency spectrograms were computed for each trial, from 0 to 1500 ms following stimulus presentation. A 2-D peak-detection algorithm was used to identify transient increases in oscillatory power. The time point of any peak occurring within the beta band (~15–25 Hz) was recorded, and the median of these time-points computed for each trial. These medians were averaged within each participant for each trial type (relevant; irrelevant) as a measure of central tendency of the probability distribution of the beta-bursts.

**Results:**

On average, between one or two beta-bursts were recorded per trial. As predicted, these occurred significantly later during behaviorally relevant trials than during irrelevant trials, in all networks, F(1,58)= 93.5, p<0.001), consistent with normal post-event beta enhancement. This effect was significantly attenuated in schizophrenia, F(1,58)=6.01, p=.017.

**Discussion:**

These findings add to the evidence that patients with schizophrenia have reduced ability to allocate attention to behaviorally relevant information. Furthermore, the demonstration of an abnormality potentially accounted for by neural modelling of top-down influence on perceptual processing opens the way to understanding the relevant neural mechanism and to developing neuromodulatory treatments that might alleviate aberrant selective attention in schizophrenia.

